# Structural Characteristics and Non-Linear Optical Behaviour of a 2-Hydroxynicotinate-Containing Zinc-Based Metal-Organic Framework

**DOI:** 10.3390/molecules20058941

**Published:** 2015-05-18

**Authors:** Shruti Mendiratta, Cheng-Hua Lee, Sih-Ying Lee, Ya-Chuan Kao, Bor-Chen Chang, Yih-Hsing Lo, Kuang-Lieh Lu

**Affiliations:** 1 Institute of Chemistry, Academia Sinica, Taipei 115, Taiwan; E-Mails: shruti.mendiratta@gmail.com (S.M.); d9722102@mail.ntust.edu.tw (C.-H.L.); pokesp@gmail.com (S.-Y.L.); ap123280510@gmail.com (Y.-C.K.); 2 Department of Applied Physics and Chemistry, University of Taipei, Taipei 106, Taiwan; E-Mail: yhlo@utaipei.edu.tw; 3 Department of Chemistry, National Central University, Taoyuan 320, Taiwan; E-Mail: bchang@ncu.edu.tw

**Keywords:** 4,4′-bipyridine, emission, 2-hydroxynicotinic acid, metal-organic framework, non-linear optics

## Abstract

Materials with non-linear optical (NLO) properties play an important role in the construction of electronic devices for optical communications, optical data processing and data storage. With this aim in mind, a Zn(II)-based metal-organic framework {[Zn_2_(nica)_2_(bpy)_1.5_(H_2_O)]·0.5(bpy)·3H_2_O}_n_ (**1**), was synthesized using 4,4ʹ-bipyridine (bpy) and a potentially bidentate ligand, 2-hydroxynicotinic acid (H_2_nica) with a salicylate binding moiety. A single-crystal X-ray diffraction analysis revealed that compound **1** crystallized in the orthorhombic space group *Fdd*2 and was composed of a three dimensional porous framework. Since *Fdd*2 belonged to a class of non-centrosymmetric space groups, we therefore investigated the non-linear optical behaviour of compound **1**. Photoluminescence studies revealed that compound **1** exhibited a blue light emission with a maxima at 457 nm.

## 1. Introduction

Metal-organic frameworks (MOFs) have gained considerable attention as a new type of multifunctional materials owing to their numerous real and potential applications in gas sorption, storage, chemical and biological detection, medical imaging and light emitting devices [1,2,3,4,5,6,7]. These hybrid materials take advantage of the properties of both traditional inorganic and organic materials and comprise novel functional materials with a degree of structural predictability [8]. Further, a non-centrosymmetric organization of molecular building blocks is essential for a bulk material to exhibit second order non-linear optical (NLO) effects and the construction of such acentric MOFs presents a great challenge to conventional synthetic strategies [9,10,11]. Non-linear optics as a bridge linking photons and electrons have gained interest due to their extensive applications in harmonic generation, amplitude and phase modulation, signal transmission, processing and storage, and promise to have a great impact on information technologies, in which NLO materials play important roles [12,13].

**Scheme 1:**
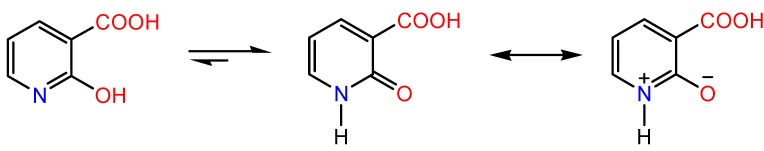
Keto-enol tautomerism in H_2_nica.

The 2-hydroxynicotinic acid (H_2_nica) ligand contains a salicylate binding moiety and can bind to metal ions by different coordination modes, namely, monodentate, bridging, *N,O*-chelation (involving the pyridine nitrogen and the oxygen in position-2, with the formation of a four membered chelate ring) and *O,O*-chelation (involving the carboxylate group and the oxygen in position-2, resulting in a six membered chelate ring). In addition, H_2_nica is characterized by keto-enol tautomerism (Scheme 1), since the labile hydrogen atom of the OH group is in very close proximity to the basic N atom and can be easily attached to it. In the solid state the ketone form is favored as it is stabilized by intramolecular hydrogen bonding between the COOH and C=O groups.

As part of our research on functional crystalline materials [14,15,16,17,18], we envisaged that the H_2_nica ligand may be suitable for use in the construction of NLO metal-organic frameworks due to its structural characteristics. Herein we report on an interesting Zn-based coordinated framework {[Zn_2_(nica)_2_(bpy)_1.5_(H_2_O)]·0.5(bpy)·3H_2_O}*_n_* (**1**, bpy = 4,4′-bipyridine). Importantly, compound **1** features: (1) preparation through a one-step self-assembly process; (2) a three dimensional porous MOF framework; (3) blue light emission with a maxima at 457 nm; (4) it crystallizes in the crystal class *mm*2 (point group *C*_2*v*_), with an orthorhombic non-centrosymmetric space group (*Fdd*2); (5) a modest second harmonic generation (SHG) intensity in comparison to SiO_2_. To the best of our knowledge, such non-linear optical behaviour and luminescence properties of such a coordination compound have never been previously explored.

## 2. Results and Discussion

### 2.1. Synthesis 

**Scheme 2:**
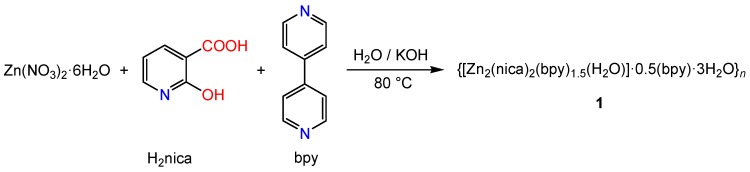
The synthesis of compound **1**.

**Scheme 3:**
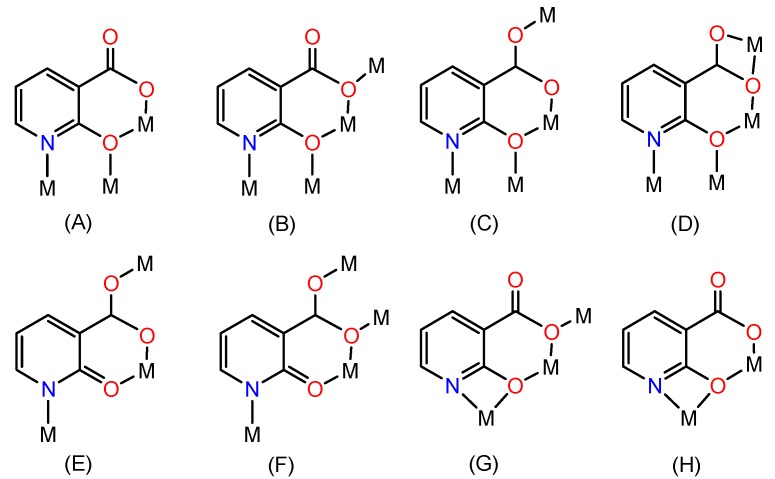
Possible coordination modes of the nica^2−^ ligand.

Compound **1** was synthesized from a mixture of Zn(NO_3_)_2_∙6H_2_O, H_2_nica and bpy via a single-step, self-organization process (Scheme 2). The FTIR spectrum of **1** showed the presence of pyridine and carboxylate groups, implying that the nica^2−^ and bpy ligands have coordinated to the metal centers. The appropriate choice of an organic ligand with a specific geometry and heteroatoms is crucial for the success of the self-assembly reaction. The H_2_nica molecule, despite its simplicity, is a very versatile bridging ligand. The chelating and partially-deprotonated nature of this ligand generates multiple coordination modes [19,20]. Scheme 3 shows the possible coordination modes of the fully deprotonated nica^2−^ ligand. More importantly, a non-centrosymmetric geometry of the final product can be generated by this ligand, including *N,O*-chelation (through the pyridine nitrogen and the deprotonated phenolate oxygen, forming a four membered chelate ring) as in modes G and H, and *O,O*-chelation (through the carboxylate group and the deprotonated phenolate oxygen, forming a six membered chelate ring, salicylate type chelation) as in modes A to H.

### 2.2. Crystal Structure

**Figure 1:**
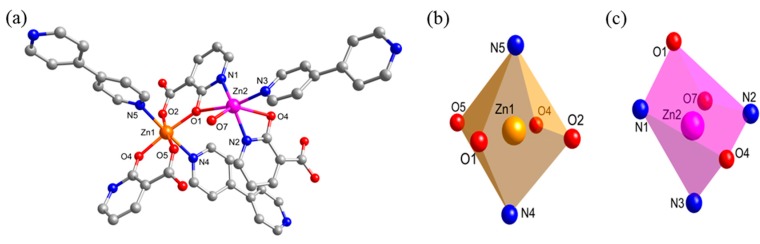
(**a**) The asymmetric unit of **1**; (**b**) view of the local coordination environment of Zn1 center; (**c**) local coordination environment of Zn2 center [symmetry code: (i): (−0.25 + x, 0.25 − y, −0.25 + z); (ii): (0.25 − x, 0.25 + y, 0.25 + z)].

A single-crystal X-ray diffraction analysis showed that compound **1** crystallized in the crystal class *mm*2 (point group *C*_2*v*_), with an orthorhombic non-centrosymmetric space group (*Fdd*2). The asymmetric unit of **1** consisted of two crystallographically distinct metal centers, two nica^2−^ ligands, one and a half bpy ligands and one coordinated water molecule. The possible coordination modes of the nica^2‒^ ligand are shown in Scheme 3 and mode H was observed in the structure of **1**. As shown in Figure 1b,c, the Zn1(II) center adopts a six-coordinated {ZnN_2_O_4_} octahedral geometry surrounded by four oxygens (O1, O2 and O4, O5) of two different nica^2‒^ ligands in a chelating mode and two nitrogens (N4 and N5) of two different bpy ligands. On the other hand, Zn2(II) center displays a six-coordinated {ZnN_3_O_3_} distorted octahedral geometry constructed by two nitrogens and two oxygens (O1, N1 and O4, N2) from the nica^2−^ ligands in a chelating mode, one nitrogens (N3) of bpy ligand, and one of the coordination water (O7). The Zn-N bond lengths are in the range of 2.038(2)–2.277(2) Å, and the Zn-O bond lengths are in the range of 1.990(2)–2.347(2) Å. The crystallographic data and structural refinements for **1** are summarized in Table 1 and the corresponding bond lengths and bond angles are listed in Tables S1 and S2 (Supplementary Materials).

**Table 1:** Summary of crystal data and refinement results.

Compound	1
chemical formula	C_32_H_30_N_6_O_10_Zn_2_
formula weight	789.36
crystal system	Orthorhombic
space group	*Fdd*2
temperature	100.0(2)
*a* (Å)	26.630(5)
*b* (Å)	35.928(7)
*c* (Å)	13.001(3)
*V* (Å^3^)	12,439(4)
Z	16
*D_calcd_* (g/cm^3^)	1.686
θ range/(°)	1.83–27.10
µ** (mm^−^^1^)	1.614
*F* (000)	6464
reflns collected	6515
unique reflns	6776
parameters	451
*R*_int_	0.0426
*R*_1_, *wR*_2_^a^ (*I* > 2σ(*I*))	0.0211, 0.0492
*R*_1_, *wR*_2_^a^ (all data)	0.0227, 0.0497
GOF	1.071

^a^ *R*_1_ = Σ||*F*_0_| − |*F*_c_||/Σ|*F*_0_|; *wR*_2_ = [Σ*w*(*F*_0_^2^ − *F*_c_^2^)^2^/Σw(*F*_0_^2^)^2^]^1/2^.

**Figure 2:**
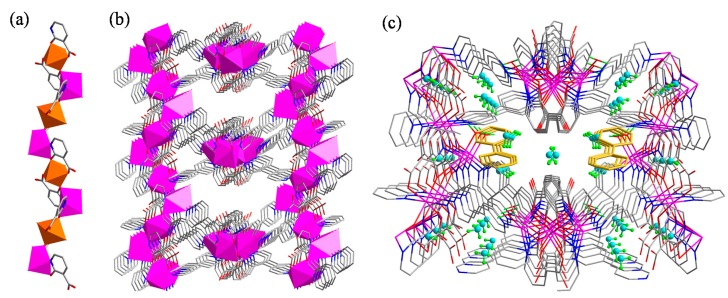
(**a**) Formation of metal chains in **1** through the coordination of nica^2‒^ ligands in a chelating manner; (**b**) the 3D framework in **1** viewed along the *c* axis; (**c**) incorporation of guest molecules such as water and 4,4′-bipyridine.

In the structure, a zig-zag chain formed through the chelation of nica^2−^ ligands with Zn(II) ions in the *N,O* and *O,O*-chelation modes can be observed along the *c* axis (Figure 2a). Further, the bpy ligands extend the one dimensional structure to a three dimensional porous framework by coordination with the neighbouring one dimensional chains (Figure 2b). Free guest molecules such as water and 4,4′-bipyridine occupy the voids in the framework (Figure 2c). Coordinated water molecules show strong hydrogen bonding interactions with the carboxylate group of nica^2−^ ligand and guest water molecules (Table S3 and Figure S1, Supplementary Materials). In addition to the hydrogen bonding interactions, strong C–H···π (purple and blue dashed lines) and relatively weaker π–π (green dashed line) interactions can also be seen between the free bpy ligands and the coordinated bpy ligands (Figure S1, Supplementary Materials). Importantly, compound **1** crystallizes in the non-centrosymmetric space group *Fdd*2 and its polar axis lies along a zig-zag chain. The polarity results from the arrangement of all of the bridging nica^2−^ ligands.

### 2.3. Thermal Stability

**Figure 3:**
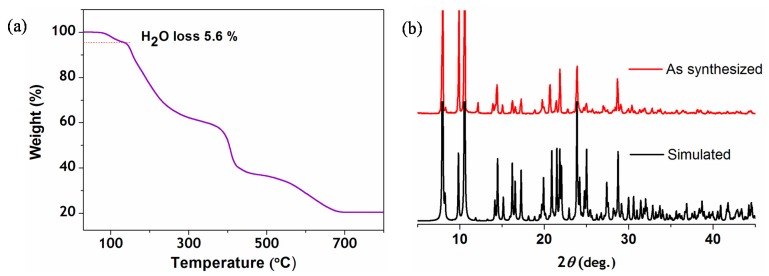
(**a**) Thermogravimetric plot of compound **1**; (**b**) the corresponding PXRD patterns of compound **1**.

To assess the thermal stability and structural variation as a function of temperature, TGA analyses of a single phase polycrystalline sample were carried out (Figure 3a). Compound **1** was found to undergo a weight loss of 5.6% at a temperature of around 140 °C, corresponding to the loss of guest water molecules. The corresponding powder X-ray diffraction pattern of **1**, agrees well with a simulated pattern (Figure 3b) and the purity of compound was further confirmed by elemental analysis.

### 2.4. Photoluminescence Studies

**Figure 4:**
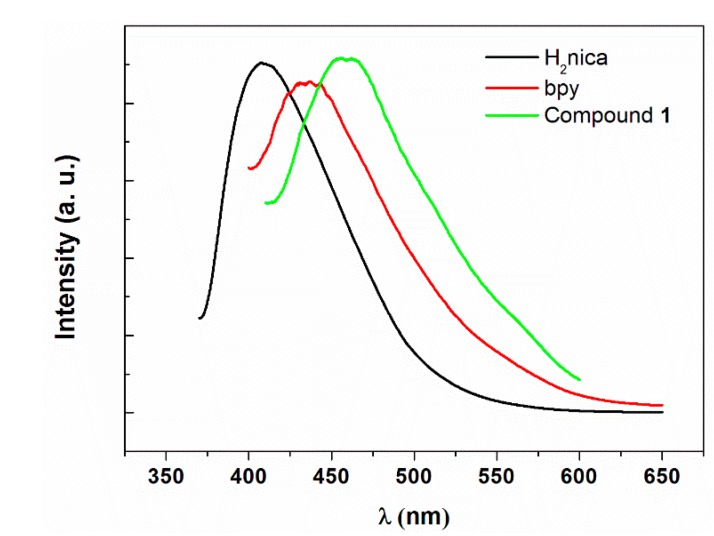
Emission spectra of **1** compared with the ligands.

The solid-state photoluminescent properties of **1** were investigated at room temperature. An emission spectrum of this compound revealed that, when excited with a wavelength of 397 nm, a blue light emission was observed at around 457 nm (Figure 4). Compared with the free ligands bpy and H_2_nica, the emission peak for **1** was significantly red-shifted. Figure showing the corresponding excitation spectra for ligands and compound **1** has been included as Figure S3 (Supplementary Materials). The emission of compound **1** can be attributed to either ligand-to-ligand charge transfer transition (LLCT) or a ligand-to-metal charge transfer transition (LMCT) or a combination of both [21,22].

### 2.5. Non-Linear Optical Studies

A number of Zn-based metal-organic frameworks and Schiff-base complexes have been investigated for their second order NLO properties [23,24,25,26]. We recently reported on the preparation of multifunctional chiral MOFs displaying low dielectric, luminescent and second order NLO properties [14]. Interestingly, Zeng and co-workers reported the inclusion of polyiodide anions in microporous MOFs and explored the kinetics of the release and recovery of iodine molecules [27]. They probed the effect of polyiodide anions on non-linear optical activity between the as-synthesized sample and the neutral iodine released sample. Inspired by the above studies we envisaged that a versatile ligand such as H_2_nica may be suitable for the construction of non-centrosymmetric MOFs.

**Figure 5:**
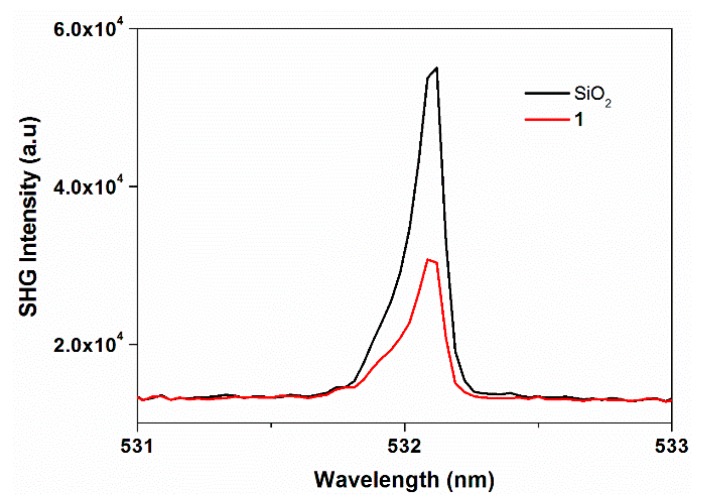
SHG plot of compound **1**.

Second-order non-linear optical effects were examined to confirm the physical properties derived from the assignment of compound **1** to a crystal class *mm*2 (point group C_2*v*_) with an acentric space group (*Fdd*2). To detect the non-linear optical properties, as per the methods recommended by Kurtz and Perry [28], the second harmonic generation (SHG) efficiency was measured on the single crystal of **1** using a laser source (Figure 5). The observed peak appeared at 532 nm and its SHG intensity was found to be weaker than SiO_2_. In theory, the SHG intensity *I_2__ω_* from any interface of the crystal in either reflection or transmission geometry is proportional to the square of the NLO coefficient χ^(2)^ and to the energy of the fundamental frequency beam *I_ω_* [Equation (1)]:

(1) $$I_{2\omega }=\frac{32\;\pi^{3}\omega^{2}s^{2}\theta_{2\omega }}{c^{3}}|e_{2\omega }\cdot \chi^{\left(2\right)}{e_{\omega }^{2}|}^{2}I_{\omega }^{2}\;\propto \;{\left(\chi^{\left(2\right)}\right)}^{2}\;I_{\omega }^{2}$$

where *θ* is the angle from the surface normal, at which the SHG signal occurs, the vectors e*_ω_* and e*_2__ω_* describe the fundamental and the second harmonic light fields at the surface [10,29]. The SHG efficiency depends on various factors such as: the donor-acceptor system in the framework, the extent of non-centrosymmetry in the system, the intensity of the push-pull dipole effect exerted by the ligand and finally the functional groups that are attached to the ligands and the guest molecules that are incorporated in the structure [10,30,31,32,33,34].

The modest SHG response in the present case can be attributed to the partial cancellation of non-centrosymmetry between the intercrossing zig-zag chains as the dipole moments are cancelled out and partly to the absorption by the crystal in the presence of bpy guest molecules.

## 3. Experimental Section

### 3.1. General Information

All chemicals were purchased commercially and were used as received without further purification. Diffraction measurements for compound **1** were carried out using a Bruker-Nonius Kappa CCD diffractometer (Bruker, Karlsruhe, Germany) with graphite-monochromated Mo-Kα radiation. The structures were solved using direct methods and refined using the SHELXS-2013 [35] program (2013, SHELX, Göttingen, Germany) by full-matrix least squares on *F*^2^ values. CCDC 1056788 contains the supplementary crystallographic data for this paper. These data can be obtained free of charge via website [36] (or from the CCDC, 12 Union Road, Cambridge CB2 1EZ, UK; Fax: +44-1223-336033; E-mail: deposit@ccdc.cam.ac.uk). Elemental analyses were conducted on a 2400 CHN elemental analyzer (Perkin-Elmer, Waltham, MA, USA). Infrared spectra were recorded in the range of 4000–400 cm^−^^1^ on a Perkin-Elmer Paragon 1000 FT-IR spectrophotometer. Thermogravimetric analyses (TGA) were performed under a nitrogen atmosphere with a Perkin-Elmer TGA-7 TG analyser. Powder X-ray diffraction patterns were recorded with a XPert-Pro diffractometer (Philips, Eindhoven, Netherlands) at 40 kV (30 mA) with Cu-Kα (λ = 1.5406 Å).

### 3.2. Synthesis of {[Zn_2_(nica)_2_(bpy)_1.5_(H_2_O)]·0.5(bpy)ʷ3H_2_O}_n_ (**1**)

An aqueous solution (2 mL) of H_2_nica (14.0 mg, 0.1 mmol) and KOH (1 M), 6 mL aqueous solution of Zn(NO_3_)_2_∙6H_2_O (29.9 mg, 0.1 mmol) and aqueous solution (6 mL) of bpy (31.3 mg, 0.2 mmol) were mixed together and then heated in the water bath at 80 °C for three days until dark orange crystals (15.3 mg) were obtained. Yield: 19.4%. Anal. Calcd (%) for C_32_H_30_N_6_O_10_Zn_2_: C, 48.69; H, 3.83; N, 10.65. Found: C, 48.63; H, 3.78; N, 10.77. IR (KBr, cm^−1^): ν = 3400 (m), 3176 (m), 1951 (m), 1929 (s), 1603 (vs), 1557 (vs), 1533 (w), 1448 (w), 1481 (vs), 1423 (m), 1408 (s), 1374 (vs), 1253 (s), 1216 (s), 1150 (s), 1096 (m), 1067 (s), 1006 (m), 991 (m), 974 (m), 953 (w), 934 (s), 795 (vs), 729 (s), 678 (m), 660 (m), 631 (vs), 590 (w), 570 (m), 542 (w), 496 (s), 474 (m) cm^−^^1^.

## 4. Conclusions

A 3D non-centrosymmetric Zn(II)-based MOF was successfully synthesized. Compound **1** crystallized in the crystal class *mm*2 (point group *C*_2v_), with an orthorhombic non-centrosymmetric space group (*Fdd*2). Although its SHG intensity was found to be modest in comparison to the traditional NLO materials, a possible structure-NLO property relationship is demonstrated. These results prompted us to synthesize MOFs with better NLO properties in the future.

## Supplementary Materials

Supplementary materials can be accessed at: http://www.mdpi.com/1420-3049/20/05/8941/s1.
