# Supplementary material for: Structural Characteristics and Non-Linear Optical Behaviour of a 2-Hydroxynicotinate-Containing Zinc-Based Metal-Organic Framework
Source: Molecules. 2015 May 18;20(5):8941–51. doi: 10.3390/molecules20058941 (PMC6272795; doi:10.3390/molecules20058941)
Supplement: Supplementary file 1 [file molecules-20-08941-s001.pdf]

Supporting Information

Table S1. Selected bond distances (Å) for **1** [a].

M–X (X=O, N)	(Å)	M–X (X=O, N)	(Å)
Zn(1)–O(4)	1.990(2)	Zn(1)–O(1)	2.005(2)
Zn(1)–O(2)	2.052(2)	Zn(1)–O(5)	2.103(2)
Zn(1)–N(5)	2.261(2)	Zn(1)–N(4)#1	2.277(2)
Zn(2)–O(7)	2.032(2)	Zn(2)–N(2)#2	2.038(2)
Zn(2)–N(1)	2.048(2)	Zn(2)–N(3)	2.065(2)
Zn(2)–O(1)	2.347(2)		

[a] Symmetry transformations used to generate equivalent atoms: #1 − x + 1/4, y + 1/4, z + 1/4; #2 x − 1/4, −y + 1/4, z − 1/4.

Table S2. Selected bond angles (°) for **1** [a].

X–M–X (X=O, N)	(°)	X–M–X (X=O, N)	(°)
O(4)–Zn(1)–O(1)	172.73(8)	O(4)–Zn(1)–O(2)	97.81(8)
O(1)–Zn(1)–O(2)	88.79(8)	O(4)–Zn(1)–O(5)	89.45(8)
O(1)–Zn(1)–O(5)	84.04(8)	O(2)–Zn(1)–O(5)	172.58(8)
O(4)–Zn(1)–N(5)	91.12(8)	O(1)–Zn(1)–N(5)	91.85(8)
O(2)–Zn(1)–N(5)	90.66(8)	O(5)–Zn(1)–N(5)	87.68(8)
O(4)–Zn(1)–N(4)#1	91.16(8)	O(1)–Zn(1)–N(4)#1	85.71(8)
O(2)–Zn(1)–N(4)#1	90.51(8)	O(5)–Zn(1)–N(4)#1	90.85(8)
N(5)–Zn(1)–N(4)#1	177.27(8)	O(7)–Zn(2)–N(2)#2	93.81(9)
O(7)–Zn(2)–N(1)	124.56(8)	N(2)#2–Zn(2)–N(1)	125.28(9)
O(7)–Zn(2)–N(3)	99.84(9)	N(2)#2–Zn(2)–N(3)	100.70(9)
N(1)–Zn(2)–N(3)	107.96(9)	O(7)–Zn(2)–O(1)	84.24(7)
N(2)#2–Zn(2)–O(1)	92.32(7)	N(1)–Zn(2)–O(1)	59.55(8)
N(3)–Zn(2)–O(1)	166.01(8)		

[a] Symmetry transformations used to generate equivalent atoms: #1 − x + 1/4, y + 1/4, z + 1/4; #2 x − 1/4, −y + 1/4, z − 1/4.

Table S3. Hydrogen bond distances (Å) and angles (°) for **1**.

D–H···A	H···A (Å)	D···A (Å)	D–H···A (°)
O(7)–H···O(5)	1.918	2.749	161.21
O(7)–H···O(10)	1.769	2.596	167.79
O(8)–H···O(3)	2.088	2.911	172.89
O(8)–H···O(6)	2.058	2.890	173.94
O(9)–H···O(6)	1.965	2.764	162.83
O(10)–H···O(3)	1.864	2.678	165.12
O(10)–H···O(9)	1.869	2.697	174.49

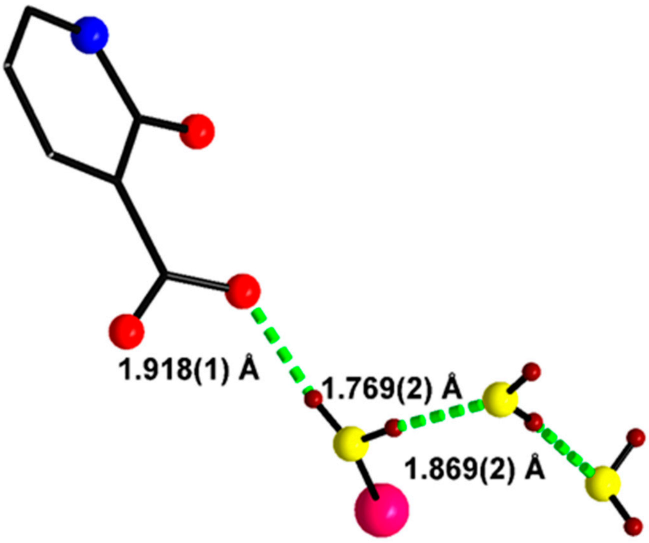

Figure S1. Hydrogen bonding interactions between the coordinated water molecules, carboxylate group of nica^{2-} ligand and guest water molecules.

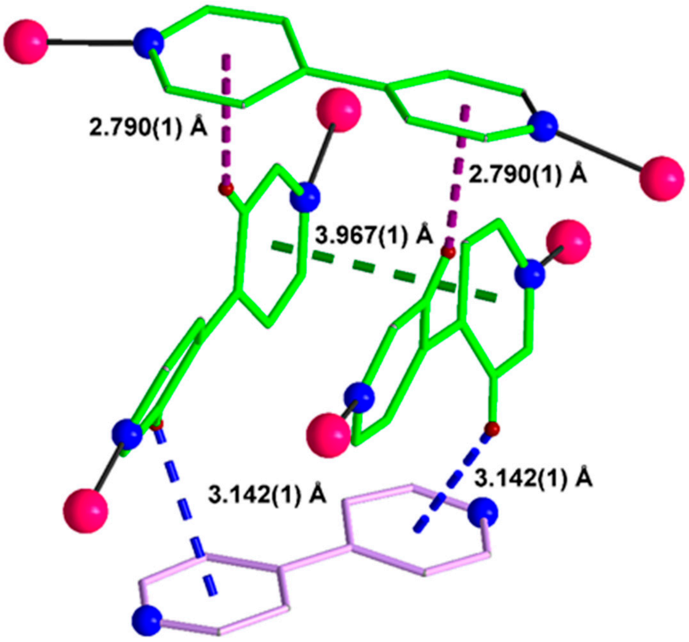

Figure S2. C–H···π (purple and blue dashed lines) and relatively weaker π–π (green dashed line) interactions are showed between free bpy ligand and the coordinated bpy ligands.

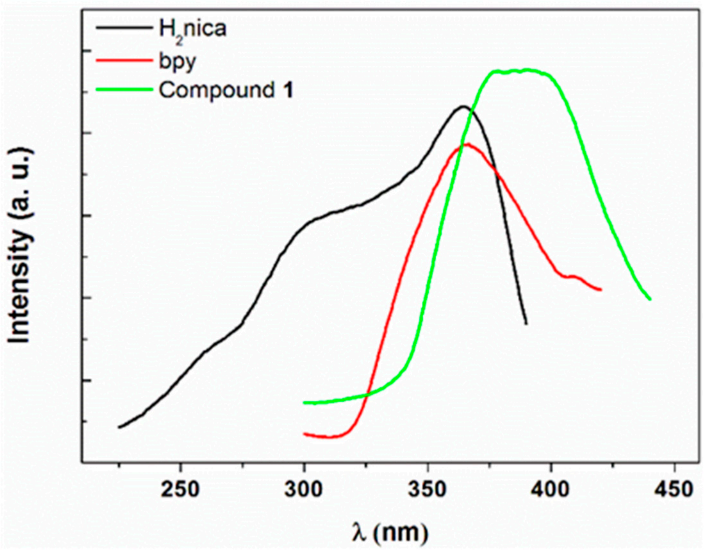

Figure S3. Excitation spectra of compound **1** compared with the ligands.
